# Facilitators for retaining men who have sex with men in pre-exposure prophylaxis care in real world clinic settings within the United States

**DOI:** 10.1186/s12879-022-07658-y

**Published:** 2022-08-05

**Authors:** Brooke G. Rogers, C. Sosnowy, A. Zanowick-Marr, P. A. Chan, L. A. Mena, R. R. Patel, W. C. Goedel, T. Arnold, C. Chu, D. Galipeau, M. C. Montgomery, K. Curoe, A. Underwood, J. Villalobos, C. Gomillia, A. S. Nunn

**Affiliations:** 1 Department of Medicine, Division of Infectious Diseases, Warren Alpert Medical School, Brown University, Providence, RI 02903 USA; 2 Department of Psychiatry and Human Behavior, Warren Alpert Medical School of Brown University, Providence, RI 02903 USA; 3 Department of Social and Behavioral Sciences, Brown University School of Public Health, Providence, RI 02903 USA; 4 Department of Population Health Science, University of Mississippi Medical Center, Jackson, MS 39216 USA; 5 Department of Medicine, Division of Infectious Diseases, University of Mississippi Medical Center, Jackson, MS 39216 USA; 6 Department of Infectious Diseases, Washington University School of Medicine, St. Louis, MO 63110 USA; 7 Department of Epidemiology, Brown University School of Public Health, Providence, RI 02903 USA

**Keywords:** Pre-exposure prophylaxis, Medication adherence, Persistence, Retention in care, PrEP continuum

## Abstract

**Background:**

Pre-exposure prophylaxis (PrEP) can significantly reduce HIV acquisition especially among communities with high HIV prevalence, including men who have sex with men (MSM). Much research has been finding suboptimal PrEP persistence; however, few studies examine factors that enhance PrEP persistence in real-world settings.

**Methods:**

We interviewed 33 patients who identified as MSM at three different PrEP clinics in three regions of the U.S. (Northeast, South, Midwest). Participants were eligible if they took PrEP and had been retained in care for a minimum of 6 months. Interviews explored social, structural, clinic-level and behavioral factors that influencing PrEP persistence.

**Results:**

Through thematic analysis we identified the following factors as promoting PrEP persistence: (1) navigation to reduce out-of-pocket costs of PrEP (structural), (2) social norms that support PrEP use (social), (3) access to LGBTQ + affirming medical providers (clinical), (4) medication as part of a daily routine (behavioral), and (5) facilitation of sexual health agency (belief).

**Discussion:**

In this sample, persistence in PrEP care was associated with structural and social supports as well as a high level of perceived internal control over protecting their health by taking PrEP. Patients might benefit from increased access, LGBTQ + affirming medical providers, and communications that emphasize PrEP can promote sexual health.

## Introduction

Pre-exposure prophylaxis (PrEP) is a highly effective, biomedical approach to HIV prevention that involves taking an oral medication once daily [1–4]. In the United States (U.S.), HIV disproportionately impacts gay, bisexual and other men who have sex with men (MSM), who represent nearly 70% of new HIV diagnoses [5].

PrEP uptake has been slow in clinical settings across the U.S [6, 7] and retention in care has been suboptimal [8–10]. In a study of 7148 individuals who initiated PrEP through a national chain pharmacy in the United States, only 56% persisted in PrEP use from initiation to end of year one, and 41% from initiation to end of year two. The lowest persistence was observed in women and individuals 18–24 years old and factors that were associated with discontinuing PrEP included financial barriers, changes in perceived risk, and difficulty accessing healthcare [11]. Data from previous clinic-based studies also show similar steep declines in use over time. In a study conducted across three U.S. cities in PrEP academic medical center affiliated PrEP clinics only 57% of MSM who initiated PrEP were retained at six months [8, 9, 11, 12]. Prior research has identified the following factors may negatively impact PrEP persistence: low risk perception [13], stigma [14–16], disruptions in daily routine and substance use [17], real and perceived side effects [14, 16, 18, 19], cost and lack of insurance coverage and financial support. [14, 16, 20, 21]

While many studies explain why people fall out of care [22], less is understood about factors that may facilitate PrEP persistence. Importantly, these factors are distinct from the inverse of barriers. For example, a barrier to using PrEP might be the cost of the medication; however, reducing or eliminating the cost of PrEP is not sufficient to support PrEP persistence over time [23]. Prior studies from clinical trials indicate that facilitators to retention in PrEP care include access to health care [24, 25], access to free PrEP [25, 26], social and emotional supports [24, 25, 27], feelings of increased safety and “peace of mind” [19, 28, 29], and positive feelings about their health [16, 30]. However, little is understood about factors that enhance PrEP persistence in real world settings. This article explores factors that have enhanced PrEP persistence among diverse MSM in three, real world clinic settings throughout the U.S.

## Methods

### Sampling and recruitment

We interviewed 33 MSM who had newly initiated PrEP at three clinics in the United States affiliated with academic medical centers in Providence, Rhode Island (RI) (n = 12); Jackson, Mississippi (MS) (n = 5); and Saint Louis, Missouri (MO) (n = 16) from October 26, 2018 to June 10, 2020. Participants were part of a larger study examining PrEP persistence over time among a large cohort of geographically, racially, and ethnically diverse MSM.

Inclusion criteria for the parent study included: assigned male at birth and identified as male (cisgender man), English or Spanish speaking, and 18 years old or older, and reported condomless anal sex with another male within the past 90 days. Individuals were eligible for an interview if they had been taking PrEP for at least 6 months (self-report), attending clinic visits, and regularly filling their PrEP prescription (verified by pharmacy records).

### Recruitment and data collection

Research staff contacted individuals via phone up to three times to invite them to be interviewed. We conducted interviews until no new data emerged and saturation had been reached [31]. Interviews lasted approximately 30–60 min and were conducted by trained research staff in person or by phone. Researchers followed a semi-structured interview guide and took notes following each interview. Interviews explored structural, social, clinical, and behavioral factors, as well as beliefs about PrEP [16]. As we describe them within this manuscript, structural factors include policies, institutions, environments, and services that impact health; clinical factors involve those directly related to clinical care experiences; social factors include experiences with other people and/or observation of others’ experiences through media; behavioral factors are related to personal actions taken to protect their health; and, beliefs are attitudes about PrEP as an HIV prevention and health promotion tool.

Interviews explored the overview of the patient experience, adherence, clinic visits, costs associated with PrEP, pharmacy and mail order experience, impact of COVID-19, social factors and discrimination, and solutions or ideas about interventions that could improve adherence for others, and thoughts on “next generation” PrEP. Interviews were digitally recorded. Recordings were professionally transcribed, de-identified, and reviewed for accuracy by study staff. Each participant received a $50 gift card as compensation for their time.

Analyses included deductive and inductive approaches to qualitative data. First, we developed a preliminary coding scheme based on the topics covered in the interview guide. Four members of the research team coded four transcripts and met twice to compare results, resolve discrepancies, and refine the coding scheme. During analysis, we used a general inductive approach to identify emergent codes, themes, patterns and conclusions from the interviews [32]. Quality checks were conducted on 20% of all transcripts and interviewer notes via iterative coding by at least two coders. Coders then wrote memos summarizing findings from transcripts. All activities were approved by the Institutional Review Boards at The Miriam Hospital in RI, University of Mississippi Medical Center in MS, and Washington University School of Medicine in MO.

## Results

Participants ranged in age from 20 to 60 years old. The sample was relatively diverse with respect to race as participants identified as White (54.5%), Black (30.3%), and Other (15.2%) races. Most had health insurance (90.9%) with most reporting private insurance (75.8%) and the rest reporting public insurance/Medicaid (15.2%). Participants were diverse with regards to education level, employment, and annual salary. Most had never taken PrEP prior to the study (72.7%). For more details on sample demographics, see Table 1.

**Table 1:** Demographic and behavioral factors among participants of adherent PrEP interviews

	Mississippi		Missouri		Rhode Island		Total	
	*n = 5*	*%*	*n = 16*	*%*	*n = 12*	*%*	*n = 33*	*%*
*Age (median, IQR)*	(26.0, 22–33)		(29.6, 20–63)		(40.3, 19–69)		(30.0, 25–34)	
*Race*								
White	1	(20.0%)	9	(56.3%)	8	(66.6%)	18	(54.5%)
Black	4	(80.0%)	5	(31.3%)	1	(8.3%)	10	(30.3%)
Asian	0	(0.0%)	1	(6.3%)	1	(8.3%)	3	(9.1%)
Other	0	(0.0%)	0	(0.0%)	1	(8.3%)	1	(3.0%)
Multiple Races	0	(0.0%)	1	(6.3%)	2	(16.7%)	1	(3.0%)
Decline to Answer	0	(0.0%)	0	(0.0%)	0	(0.0%)	0	(0.0%)
*Ethnicity*								
Hispanic/Latino	0	(0.0%)	0	(0.0%)	1	(8.3%)	1	(3.0%)
Non-Hispanic/Latino	5	(100.0%)	16	(100.0%)	11	(91.7%)	32	(97.0%)
*Sexual orientation*								
Gay	4	(80.0%)	15	(92.7%)	11	(91.7%)	30	(90.9%)
Bisexual	1	(20.0%)	1	(6.3%)	0	(0.0%)	2	(6.1%)
Queer	0	(0.0%)	0	(0.0%)	1	(8.3%)	1	(3.0%)
*Health insurance*								
Private	4	(80.0%)	12	(75.0%)	9	(75.0%)	25	(75.8%)
Medicare	0	(0.0%)	0	(0.0%)	0	(0.0%)	0	(0.0%)
Medicaid	0	(0.0%)	2	(12.5%)	3	(25.0%)	5	(15.2%)
None	1	(20.0%)	2	(12.5%)	0	(0.0%)	3	(9.1%)
*Education level*								
Some high school	0	(0.0%)	0	(0.0%)	0	(0.0%)	0	(0.0%)
High school graduate	0	(0.0%)	0	(0.0%)	2	(16.7%)	2	(6.1%)
Some college/technical school	2	(40.0%)	5	(31.2%)	2	(16.7%)	9	(27.3%)
College graduate	2	(40.0%)	8	(50.0%)	4	(33.3%)	14	(42.4%)
Graduate School	1	(20.0%)	3	(18.8%)	4	(33.3%)	8	(24.2%)
*Employment status*								
Full-time	3	(60.0%)	11	(68.8%)	9	(75.0%)	23	(69.7%)
Part-time	1	(20.0%)	3	(18.8%)	1	(8.3%)	5	(15.2%)
Unemployed	0	(0.0%)	1	(6.3%)	1	(8.3%)	2	(6.1%)
Student	1	(20.0%)	0	(0.0%)	0	(0.0%)	1	(3.0%)
Retired	0	(0.0%)	0	(0.0%)	1	(8.3%)	1	(2.0%)
Other	0	(0.0%)	1	(6.3%)	0	(0.0%)	1	(2.0%)
*Annual income*								
Less than $10,000	0	(0.0%)	1	(6.3%)	0	(0.0%)	1	(3.0%)
$10,001 to $20,000	1	(20.0%)	1	(6.3%)	0	(0.0%)	2	(6.1%)
$20,001 to $50,000	1	(20.0%)	6	(37.5%)	2	(16.7%)	11	(33.3%)
$50,001 to $75,000	2	(40.0%)	2	(12.5%)	4	(33.3%)	8	(24.2%)
$75,001 or more	1	(20.0%)	3	(18.8%)	5	(41.7%)	9	(27.2%)
Declined to answer	0	(0.0%)	1	(6.3%)	1	(8.3%)	2	(6.1%)
*Ever taken PrEP*								
Yes	2	(40.0%)	4	(25.0%)	2	(16.7%)	8	(24.2%)
No	3	(60.0%)	12	(75.0%)	9	(75.0%)	24	(72.7%)

Themes identified through analysis included structural, clinical, social, behavioral factors, and beliefs that were salient in helping individuals persist in PrEP care. Analyses identified themes and then classified them according to the level at which they affected PrEP persistence. Specifically, the following themes were identified: (1) navigation to reduce out-of-pocket costs of PrEP (structural), (2) social norms that support PrEP use (social), (3) access to LGBTQ + affirming medical providers (clinical), (4) medication as part of a daily routine (behavioral), and (5) facilitation of sexual health agency (belief). Below, we provide information about each of these areas as well as illustrative quotes. No notable differences were observed among recruitment sites.

### Structural factors: navigation to reduce out-of-pocket costs of PrEP

Participants noted the importance of having support from a clinic employee to help navigate the medical system, insurance, and cost of PrEP. Individuals noted that either a low monthly co-pay or using an industry supported co-pay card which eliminated co-pays altogether were helpful in making PrEP an affordable and reasonable option for their health.

One individual noted, the co-pay card significantly reduced his financial burden because his insurance costs were extremely high:

“I think my monthly payment, even through my insurance, was supposed to be 1,500 a month or something. It was crazy high, so they gave me a coupon. I don’t pay one thing for PrEP, and all my lab work has been reimbursed by my insurance, so I haven’t paid one penny.” White, 53 years old

Having PrEP as an affordable option was identified a significant factor in whether people initiate and maintain PrEP use over time:

“I think the financial aspect is probably the biggest thing for people ’cause whenever things cost money, people don’t wanna spend the money. It’s like that’s the biggest thing.” White, 34 years old

### Social factors: social norms that support PrEP use

Participants were highly engaged in LGBTQ + community in person and online and shared that PrEP was normalized and promoted in these social spaces, which reduced stigma and enhanced support for engagement and persistence in PrEP care. For example, one participant shared their observations and participated in several conversations about PrEP that occurred in an LGBTQ + Facebook group:

“I noticed that Several of the guys have posted things like, “Hey, I’m considering taking PrEP,” or “My doctor has suggested the other PrEP medication, not the Truvada. Anybody taking any of these?” It’s been really interesting to watch guys post.” Black, 34 years old

Another participant stated that observing PrEP status as part of a background on dating apps for sexual minority and other men who have sex with men helped to normalize PrEP:

“I think, honestly, at least in my perspective, it’s becoming more common to see on the LGBTQ + dating apps that people are taking it, and I think seeing it become common is also seeing it become more accepted, and it’s less of a strange idea…more people talking about PrEP, and PrEP is showing up in more places… seeing it more places is helping it become more normal, and if it becomes more normal, then people won’t feel so taboo by taking it.” White, 24 years old.

### Clinical factors: access to LGBTQ + affirming medical providers

Individuals who persisted on PrEP were proactive in engaging with their providers and initiating conversations that addressed concerns about the safety of antiretroviral medication.

One participant shared that knowing their provider would be consistent allowed them to feel comfortable talking about information:

“I was a little nervous going in at first, but meeting with Dr. <Name> and knowin’ that she’s my constant doctor that I’ve been able to be open with her and more comfortable talking to her about this information.” Black, 29 years old

Another participant shared he had some concerns about the medication impacting his kidney functioning and was able to ask the provider directly, who assured him that his kidney values would be monitored as part of routine care and that these side effects were relatively uncommon:

“The only concern I really had about taking the medication was I had heard of some kidney function problems…The doctor said that he would make sure that that wasn’t the case by checking...he alleviated my concerns by letting me know that there were very, very few cases of people having any kidney problems while taking the medication.” White, 53 years old

### Behavioral factors: medication as part of a daily routine

Participants shared the ways in which they had integrated taking PrEP into their daily routines including placing the medication bottle in the bathroom near the sink and doing it as part of their morning routines. Including PrEP as part of these routines was a strategy that helped them continue taking the medication daily:

“Get up in the morning, brush my teeth, wipe my face, take my medicine. It’s all right there.” Black, 20 years old

Another shared that in addition to morning routines, getting a pill kit helped him stay organized and remember whether he had taken the medication that day:

“I kept it in the bathroom right by my toothbrush, so when I would get up in the morning to brush my teeth, I would see the Truvada, so I’d be like, “Oh, take my medicine,” and I would just take that. Either before or after I brushed my teeth, I would do that. Then what really even helped better because occasionally I would forget, but not usually, but what really helped was I got one of those pill kits.” White, 37 years old

Consistency in taking the medication daily was key. Even when participants were not “at-risk for HIV” based on their current behavior, they persisted in continuing to take PrEP as part of their daily routine:

“Even COVID, where I am pretty much practicing very strict isolation right now, given the circumstances, I’m still takin’ it on the daily just to keep that cadence going.” White, 34 years old

### Beliefs: PrEP facilitates sexual health agency

Interestingly, many participants shared that they felt taking PrEP had promoted positive sexual health agency. Individuals reported more regular engagement in sexual health care including STI/HIV testing and treatment and increased assertiveness and pleasure in sexual relationships.

As one individual shared, being on PrEP helped him attend more medical visits. At each of these PrEP care visits, the participant was also screened for HIV and STIs, contributing to feeling more in control of his health:

“I started taking PrEP for a way to have more control of my sexual health. Just taking PrEP, yes, it does—decreases my chances of getting HIV, but also, I am able to come in more, get my bloodwork done and everything, and it has really helped me have a better control of my sexual health altogether.” Black, 20 years old

Taking PrEP also helped participants take ownership over their health and directly translated into increased self-agency. Participants also noted that others, including sexual partners, largely responded positively to learning they were taking PrEP. Participants saw it as a way of showing self-respect:

**“**You supporting your body. You lovin’ your body and knowin’ your self-worth basically. It’s really all about self-worth.” Black, 22 years old

Participants also acknowledged that PrEP increases their enjoyment of sex because they are less worried about contracting HIV:

“People that are actually getting on PrEP and basically taking PrEP to have pleasure with their sexuality.” Black, 55 years old

## Discussion

Structural, social, clinical, and behavioral factors as well as beliefs about PrEP influenced persistence in our sample. Our participants agreed that out-of-pocket costs for PrEP were important and several noted that office visits, laboratory testing, and co-pays needed to be considered in addition to the costs of medication alone. Our participants were able to benefit from navigation to help them in reducing the cost of PrEP medication and associated visits and laboratory fees, which was important for helping them continue to stay engaged in care (structural). Participants also found persons who were supportive of PrEP use both in their friendships and romantic relationships, which continued to be an important factor in them staying on PrEP (social). Participants noted that having providers who affirmed their sexual identities and were knowledgeable was also key as they felt comfortable asking questions about medication efficacy and side effects (clinical). Participants had found ways to incorporate taking PrEP into their daily routines making it easier to adhere to daily medication (behavioral); and, many found that taking PrEP enhanced their sexual health agency leading to higher rates of STI/HIV screening, assertiveness with romantic partnerships, and increased satisfaction (beliefs). Findings suggest that access to affordable medication and care were key factors in PrEP persistence and continued engagement with the care continuum.

Addressing structural factors through strategies including removing barriers to insurance coverage and reducing out-of-pocket costs pose great potential to enhance PrEP care. A prior study examined the role of insurance and found that individuals who had insurance were almost four times more likely to engage in PrEP care [21]. In a multivariate logistic regression model adjusted for age, race, education, and income, having individual insurance was the only factor significantly and positively associated with PrEP utilization [21]. Although pharmaceutical company sponsored programs or co-payment cards, like those used by participants interviewed in our study, are effective at bridging the gap for individuals in our sample, ongoing structural gaps in our current medical system may impede access to care even when these programs are available [33, 34]. Access to care remains a challenge for many due to the associated costs of medication (current list price ~ $2000 per month), provider visits, and lab testing [21]. This is further complicated by issues/gaps in insurance coverage and high levels of cost sharing even among those who are insured [35, 36]. As of July 2021, the Departments of Health and Human Services, Labor, and the Treasury issued new guidance on PrEP coverage under the Affordable Care Act [37]. The new federal guidance requires health insurance companies to cover PrEP medication and the support services without cost sharing. This change aims to reduce out-of-pocket costs and increase PrEP access for individuals at high-risk [38]. Efforts to make PrEP more affordable through legislation to ensure its coverage under current private insurance plans and limit deductibles and other out-of-pocket costs to access PrEP might help to promote PrEP persistence [35]. Government plans like Medicare, Medicaid, and state-based plans could also help provide coverage and limit out-of-pocket costs. Finally, expansion of Medicaid and other safety net insurance programs can help provide consistent access to care even as people change places of employment or romantic relationships.

However, providing increased insurance access and reduced costs may not fully address issues with clinical PrEP persistence. Poor persistence in PrEP care has been observed even in research studies when costs of clinical care and support services were fully covered [39], suggesting that factors beyond access and cost are associated with PrEP discontinuation and that, in order to improve PrEP persistence, other factors will need to be leveraged [21, 40]. These may include some of the other issues that arose in our interviews including the need for culturally informed and affirming care, normalization of PrEP use to reduce stigma, promoting behavioral strategies to remember to take the medication, and leveraging the ways that PrEP can increase sexual agency and pleasure.

Culturally tailored (LGBTQ +) clinical services may enhance PrEP persistence. Having providers and clinical staff who are non-judgmental, LGBTQ + affirmative, and helpful can also help reduce anxiety or concerns about accessing PrEP care and improve the healthcare experience for the person seeking care making it more likely they will be retained in care [19, 41]. Additionally, increased social norms around taking PrEP was perceived as important to promote persistence in care. Participants noted that the normalization of PrEP within their social circles supported their perception and ongoing adherence to PrEP. Social support has been noted as a facilitator for PrEP retention among MSM and was found to significantly impact adaptive behavioral responses, such as adherence to PrEP-related medical care, enhanced resilience to stress, and reduced impact/internalization of stigma [42–44]. Participants included in this sample were successful in remaining engaged in care and adherent to daily oral PrEP. When asked about what helped them, they shared that they found ways to incorporate PrEP into their daily schedules, which is a strategy that has been shown effective for antiretroviral adherence [45, 46]. Finally, several participants noted that taking PrEP improved their sexual health agency. Other qualitative studies of PrEP use among MSM have found similar relationships with positive psychological benefits of taking PrEP [17]. The importance of self-efficacy in condom use among MSM has been noted in prior research, and similarly, feeling confident about the ability to promote one’s own health and well-being was relevant for consistent PrEP use. [47, 48] Importantly, participants in our study reported that taking PrEP was not only associated with reduced concerns about acquiring HIV, but also with increased feelings of control over their health and self-worth, which led to meaningful and healthy sexual relationships and romantic partnerships. Much of the messaging around PrEP has been focused on clinical outcomes and HIV prevention; however, promotion of well-being, positive sexuality, and meaningful partnerships are also important parts of psychological and social health that should not be overlooked when discussing benefits of PrEP persistence with patients. MSM in our sample did not report an increase in anonymous or casual sexual partners, but instead, reported similar or even decreased sexual risk as a result of entering monogamous partnerships [26, 49]. Results from the iPrEx trial also failed to find any significant increase in sexual risk following initiation of PrEP [50]. A secondary study of the U.S. PrEP Demonstration Project, which conducted a qualitative examination of counselor notes also noted that taking PrEP was not associated with a reduction or abandonment of other sexual health practices, suggesting that PrEP could be part of a comprehensive sexual health plan and even increase positive sexual health, wellness, and pleasure. [28]

### Limitations

Our study is subject to several limitations. This sample was recruited from within PrEP clinics affiliated with academic medical centers and may not be reflective of samples who do not have access to PrEP care [51]. Additionally, although efforts have been made to engage a geographically, racial/ethnically, and otherwise diverse sample, almost all of the sample identified as gay and had higher educational achievement than perhaps the general population of individuals who are PrEP eligible. Additionally, the sample skewed older and younger MSM on PrEP may have differing views. However, the population from which this sample was drawn was relatively diverse and this sample was representative of the individuals who were persistent in PrEP care in our cohort. Additionally, this sample was largely reflective of individuals who are persistent in PrEP care in the U.S. suggesting that these individuals may be more likely to have an affirmed LGBTQ+  identity, higher education and income, and older age than those MSM who are not persistent in PrEP care.

## Conclusion

This study enhances our understanding of the lived experiences MSM retained in PrEP care and the factors that help support them in making decisions to prevent HIV and support their health from real-world experiences of PrEP patients. These findings highlight the need for addressing health systems factors like out-of-pocket costs, culturally affirming LGBTQ + medical care, supportive social norms, and sexual health agency and empowerment in promoting PrEP persistence over time. Future research and practice should continue to engage HIV affected communities and PrEP patients in conversations about how to best develop approaches to HIV prevention and PrEP care that support retention in care over time.

## Data Availability

The datasets generated and/or analysed during the current study are not publicly available, but are available from the corresponding author on reasonable request.
